# Effects of Dietary Supplementation with Mulberry (*Morus alba* L.) Leaf Polysaccharides on Immune Parameters of Weanling Pigs

**DOI:** 10.3390/ani10010035

**Published:** 2019-12-23

**Authors:** Xiangjie Zhao, Rongling Yang, Yanhong Bi, Muhammad Bilal, Zheshi Kuang, Hafiz M. N. Iqbal, Qiulan Luo

**Affiliations:** 1 College of Life Science and Food Engineering, Huaiyin Institute of Technology, Huaian 223003, China; zhaoxiangjie@hyit.edu.cn (X.Z.); biyh@hyit.edu.cn (Y.B.); bilaluaf@hotmail.com (M.B.); 2 Sericultural and Agri-Food Research Institute, Guangdong Academy of Agricultural Sciences, Guangzhou 510610, China; kuangzs123@163.com (Z.K.); qiulanluo@163.com (Q.L.); 3 Tecnologico de Monterrey, School of Engineering and Sciences, Campus Monterrey, Ave. Eugenio Garza Sada 2501, Monterrey, N.L., CP 64849, Mexico; hafiz.iqbal@tec.mx

**Keywords:** mulberry leaf, polysaccharides, immune parameters, weanling pigs, immune organ weight, immunoglobulins mRNA expression

## Abstract

**Simple Summary:**

The increasing overuse/misuse of restricted antibiotics in livestock and poultry production has raised a serious health concern. Thus, aiming to improve animals’ health levels, researchers are redirecting their focus onto naturally occurring bioactive ingredients from plants; these compounds have become a potential substitute for antibiotics. Mulberry leaf polysaccharide (MLP) is an important bioactive component, which has notable potential for improving animal immunity. In this context, the present study was designed to investigate the MLPs’ effect on animals’ metabolisms and the immune parameters of weanling pigs.

**Abstract:**

In this study, the effect of dietary supplementation of mulberry leaf polysaccharides (MLPs) on the immune parameters—i.e., the immune organ weight, serum immunoglobulins, cytokines, nitric oxide (NO) production, and insulin-Like growth factor-1 (*IGF1*) mRNA expression—of weanling pigs as a model animal was investigated. A total of 120 healthy weanling pigs (aged 28 ± 2 d) with the same body weights were randomly divided into four groups: (1) Control treatment (CT), basal diet (BD), (2) MLP low-dose treatment (MLT), 0.6 g/kg MLP + BD, (3) MLP high-dose treatment (MHT), 1.2 g/kg MLP + BD, and (4) antibiotic treatment (AT), 0.15 g/kg chlortetracycline + BD. The results revealed that the thymus and spleen indices were significantly increased (*P* < 0.05) in both MLT and MHT groups in comparison with the CT group, while the serum levels of immunoglobulin G (IgG), interleukin (IL)-1*β*, IL-2, IL-8, and interferon (IFN-*γ*) in the MLT group and IL-2, IL-6, and IFN-*γ* in the MHT group were also considerably greater (*P* < 0.05) than the corresponding levels in the CT group. The serum contents of IgG, IL-1*β*, IL-2, and IL-8 in the MLT group and IL-2 and IL-6 in the MHT group were significantly increased in comparison with the corresponding contents in the AT group (*P* < 0.05). The transformation rate of lymphocytes in the MLT and MHT groups was higher compared to the CT and AT groups. However, a notable difference was found between the MLT group and the two control groups. The peripheral lymphocyte NO production in the MLT, MHT, and AT groups was significant relative to the CT group. The expression levels of *IGF1* mRNA in the liver and muscle longissimus tissues of both the MLT and MHT groups showed significant improvement (*P* < 0.05) over those in the CT group. Moreover, the *IGF1* mRNA expression in the muscle longissimus from the MLT group was significantly higher than in the AT group. In conclusion, the results suggest that incorporating MLPs into the diets of weanling pigs improves the animals’ metabolisms and immune functions, and the effects of the MLT group were superior to those of both the MHT and AT groups.

## 1. Introduction

In the global intensive pig production sector, early weaning of piglets is a key technology. Overall, it has several advantages, such as the improvement of the sow’s reproductive performance, the reduction of the chance of disease spread from sow to piglet, the increase of the production performance of piglets, and the carcass quality [1]. However, the incompletely developed immune system and stresses (environment, diet, psychology, etc.) after weaning easily result in stagnated piglet growth and development, weakened immunity, and increased mortality and morbidity [2]. In turn, this severely affects the economic benefits of pig production. Therefore, alleviation of weaning stress and improvement of the growth, feed efficiency, and immunity of early weaning piglets is one of the research hotspots in the animal field. Antibiotics have played an essential role in animal production, disease control, and animal growth promotion [3,4]. However, concurrent security issues concerning the overuse/misuse of antibiotic-based feed additives have drawn a great deal of research attention. Recently, studies on using plant polysaccharides to replace antibiotics as livestock immunity enhancers and metabolic modulators have become an emerging global trend [5,6,7].

Polysaccharides are a class of natural macromolecules composed of more than 10 monosaccharides connected by a glycosidic bond. Based on the type and concentration, plant-based naturally occurring bioactive molecules play a vital role in numerous biological activities and biomedical applications [8,9]. In addition, the dietary supplementation of plant polysaccharides can significantly promote an animal’s immune function, immune organ development, lymphocyte activation, antibody production, and growth performance, as well as causing a reduction of animal mortality [8,9,10]. Mulberry leaf polysaccharides (MLPs) are a natural active product extracted from mulberry leaves, which possess a variety of medicinal effects and biological functions. Reports have shown that MLPs display antioxidant [11], immunomodulatory [12], and antibacterial activities [13]—among other biological functionalities—with few toxic side effects to the body. To date, there have been few reports on the use of MLPs to improve the growth performance and immune responses of weanling pigs. Therefore, the present study investigated the effects of MLPs on weanling pigs by measuring their growth performance, immune organ index, serum immunoglobulin and cytokine levels, peripheral lymphocyte proliferation, and NO production, as well as *IGF1* mRNA expression.

## 2. Materials and Methods

### 2.1. Procurement and Composition of MLPs

The MLPs were purchased from Xi’an Sinuote Bio-Tech Co., Ltd. (Xi’an, China). According to the supplier guide information sheet, as received, MLPs were brown with a powder appearance. Also, as received and used, MLPs were 92.22% pure and consisted of glucose, mannose, arabinose, galactose, xylose, rhamnose, and ribose, at the ratio of 250:66:6:3.25:2.5:1.25:1, respectively.

### 2.2. Experimental Design and Ethical Statement

A total of 120 healthy Duroc–Landrace–Yorkshire crossbred weanling pigs (aged 28 ± 2 d) with the same body weight were randomly divided into 4 groups, i.e., (1) control treatment (CT), basal diet (BD), (2) MLP low-dose treatment (MLT), 0.6 g/kg MLP + BD, (3) MLP high-dose treatment (MHT), 1.2 g/kg MLP + BD, and (4) antibiotic treatment (AT), 0.15 g/kg chlortetracycline + BD. Each group was comprised of 30 pigs. Pigs were each randomly assigned to one of the pens, with a total of 5 pens per group and 6 piglets in a pen. The investigational basal diet was designed according to the nutritional needs of pigs established by the National Research Council [14] and the feeding standard of China for weanling pigs [15]. Its nutrient levels and chemical composition are summarized in Table 1. The representative ethical committee of the Huaiyin Institute of Technology, China approved this study according to the requirements for Animal Care and Ethical Conduct.

### 2.3. Feeding Management and Sampling

Experimental trials were carried out in a closed pig house, and the animals were given free access to feed and water. The pig houses were kept clean, and the piglets all had a healthy status. The adopted immunization procedures were based on the herd’s immune status and the epidemic season of infectious diseases in combination with local specific conditions—for example, swine fever and hand, foot, and mouth disease. Animal epidemic prevention systems were implemented and supervised by the Administration of Quality Supervision, Inspection, and Quarantine of the PRC (China). Therefore, experimental animals were all negative for relevant infections (Porcine circoviruses, PCV2; Porcine reproductive and respiratory ayndrome, PRRSV; *Clostridia*, *Actinobacillus* pleuropneumonia, APP). The pre-feeding and the experimental period were 7 d and 21 d, respectively. The fasting weight of each experimental animal was noted in the morning at 0 and 21 d. The food intake per pigpen was also documented every day. All data were used for calculating the average daily gain (ADG), average daily feed intake (ADFI), and the feed to grain (F/G) ratio. The following Equations (1)–(3) were used to record the ADG, ADFI, and F/G ratio, respectively.

ADG = Total weight gain/the days of experiment(1)

ADFI = Total feed consumption/the days of experiment(2)

F/G = ADFI/ADG(3)

After the experiment, one randomly nominated pig from each pigpen was slaughtered, and the blood sample was collected by jugular venipuncture and placed into 2 centrifuge tubes (10 mL). The blood sample in the tube with 0.8 mL heparin (250 U/mL) was mixed thoroughly and stored in the refrigerator at 4 °C until its later use. The blood sample without anticoagulant in the other tube was placed at room temperature for 20–30 min and centrifuged (at 1000× *g* for 15 min). Following centrifugation, the collected serum was aliquoted and stored at −78 °C until its later use. The immune organs, i.e., thymus and spleen, were collected from the abdomen of the slaughtered piglet. After removing the attached fat tissues, the immune organs were weighed on an electronic scale, and the immune organ index was then calculated using the formula given below [16]:

Immune organ index (g/kg) = organ weight (g)/body weight (kg).

The liver and muscle longissimus tissues were placed into a 10 mL sterile tube. Immediately thereafter, liquid nitrogen was poured into the tube and preserved at −78 °C. The freshly prepared samples were used for gene expression analysis.

### 2.4. Immune Parameters Analysis

#### 2.4.1. Determination of Serum Levels of Cytokines and Immunoglobulins

Serum concentrations of interleukin-2 (IL-2), IL-6, IL-8, IL-1*β*, and interferon (IFN-*γ*) were recorded using respective radioimmunoassay kits purchased from Nanjing Jiancheng Bioengineering Institute. Serum Igs (IgG and IgM) were measured using respective IgG and IgM kits (Beijing Leadman Biochemistry Co., Ltd., Beijing, China) on the immune biochemical analyzer (Beckman Coulter, Inc, Brea, CA, USA).

#### 2.4.2. Peripheral Blood Lymphocyte Proliferation and Nitric Oxide Production

An earlier reported method was adopted to study the peripheral blood lymphocytes [17,18], with slight modifications. Briefly, the lymphocytes isolated from blood were diluted to 2 × 10^6^ cells/mL using an RPMI-1640 medium (Gibco, Grand Island, NY, USA), and cultured in 96-well tissue culture plates for 48 h at 37 °C in a 5% CO_2_ incubator. After the designated incubation, 20 μL MTT (Sigma, St. Louis, MO, USA) was added into each well, incubated (4 h), and 150 μL of dimethyl sulfoxide was incorporated into each well and shaken until complete dissolution of the precipitate. Light absorbance serving as an index of lymphocytes transformation was recorded at 570 nm using an Enzyme-linked Immunosorbent Assay Reader (Gibco, Grand Island, NY, USA). The concentration of Nitric Oxide (NO) in the supernatant of peripheral lymphocytes isolated from the blood of weaning pigs was determined using the Küskü-Kiraz et al. [19] method by measuring at 550 nm. The standard curve for nitrite was performed in a culture medium by using serial dilutions of sodium nitrite.

#### 2.4.3. The mRNA Abundances of *IGF1* in Liver and *Longissimus dorsi*

The total RNAs were extracted for the tissue samples from the liver and longissimus dorsi with TRIzol^®^ Reagent (ThermoFisher, Waltham, MA, USA), following the manufacturer’s instructions. Fluorescent real-time PCR determined the *IGF1* gene expression in liver and longissimus dorsi of weaning pigs. Amplification of PCR was performed using Taq DNA polymerase, as reported earlier [20] using the forward primer (5′-CACATCACATCCTCTTCGCA-3′) and the reverse primer (5′-CTGGAGCCGTACCCTGTG-3′). Beta-Actin (*β*-Actin), a housekeeping gene, served as an external control gene. The forward (5′-CTGCGGCATCCACGAAACT-3′) and the reverse primer (5′-GTGATCTCCTTCTGCATCCTGTC-3′) were designed to amplify the *β*-Actin gene according to the published sequence. Thermal cycling (ABI 7500 Real-Time PCR instrument (ABI, Carlsbad, CA, USA)) was performed using the following conditions: 50 °C for 2 min, 95 °C for 1 min, and 38 cycles at 95 °C for 15 s, 59 °C for 15 s, 72 °C for 40 s, and a final extension at 72 °C for 10 min. The relative quantification of gene expression changes was calculated with the positive control (*β*-Actin) by using the 2 ^(−ΔΔ*C*t)^ method [21,22].

### 2.5. Data Analysis

All data obtained in this study are reported as means of replicates and standard errors of means (mean ± S.E.). The obtained results were statistically analyzed using one-way analysis of variance (ANOVA) with the SPSS 17.0 program (SPSS Inc., Chicago, IL, USA). The difference among treatments was compared using Duncan’s multiple range tests when a different effect was observed in the experiment. A *P*-value of less than 0.05 denoted a statistically significant difference.

## 3. Results

### 3.1. Effects of MLPs on the Growth Performance and Immune Organ Weights of the Early Weaning Piglets

Table 2 summarizes the changes in ADG, ADFI, and F/G of the weanling pigs. The ADFIs in the MLT (0.6 g/kg) and MHT (1.2 g/kg) groups were not significantly different (*P* > 0.05) from those in the CT group. While the ADG in the MLT group was significantly higher (*P* < 0.05) than that in the CT and AT groups, the ADG in the MHT group did not show a significant difference (*P* > 0.05) compared with that in the CT and AT groups. The F/G in the MLT group was considerably lower than that in both the CT and AT groups; it decreased by 5.63% and 3.90%, respectively. Similarly, no significant difference was recorded in the F/G between the MHT and CT groups. As shown in Table 2, both the thymus and spleen indices in the MLT and MHT groups were noticeably greater (*P* < 0.05) than those in the CT group, but the difference between the MLT and MHT groups was not significant.

### 3.2. Effects of MLPs on the Serum Immunoglobulins and Cytokines of Weanling Pigs

As displayed in Table 3, the serum concentrations of IgG, IL-1*β*, IL-2, IL-8, and IFN-*γ* in the MLT group and the serum levels of IL-2, IL-6, and IFN-*γ* in the MHT group were significantly higher (*P* < 0.05) than the corresponding levels in the CT group. In addition, the serum contents of IgG, IL-1*β*, IL-2, and IL-8 in the MLT group and the serum levels of IL-2 and IL-6 in the MHT group were also statistically greater (*P* < 0.05) than the corresponding concentrations in the AT group.

### 3.3. Effects of MLPs on the Lymphocyte Proliferation and NO Production of Weanling Pigs

**Figure 1:**
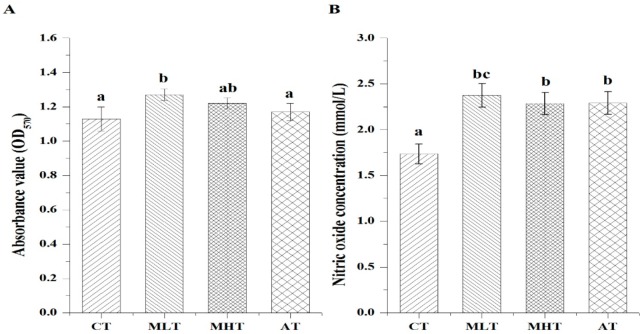
Effects of MLPs on the proliferation (**A**) and NO production (**B**) of lymphocytes from weanling pigs. Vertical bars represent standard errors (*N* = 5, samples were from one pen of each treatment). Values are expressed in means. Mean values with different letters (^a, b, c^) are significantly different within a cluster of bars, not across clusters of bars (*P* < 0.05).

Dietary supplementation of MLPs increased the lymphocyte transformation rate of weanling pigs. The lymphocyte transformation rates were higher in both the MLT and MHT groups compared with the CT and AT groups, and the differences between the MLT group and the CT and AT groups were significant (*P* < 0.05). The lymphocyte transformation rates in the MLT and MHT groups were increased by 12.39% and 7.96%, and 8.55% and 4.27%, respectively, compared with the CT and AT groups (Figure 1A). The peripheral lymphocyte NO production in the MLT, MHT, and AT groups was higher relative to the CT group. The peripheral lymphocyte NO production in the MLT and MHT groups was increased by 36.83% and 31.70%, respectively, compared with the CT group, but was not significantly different (*P* > 0.05) from that in the AT group (Figure 1B).

### 3.4. Effects of MLPs on the Expression Levels of IGF1 mRNA

**Figure 2:**
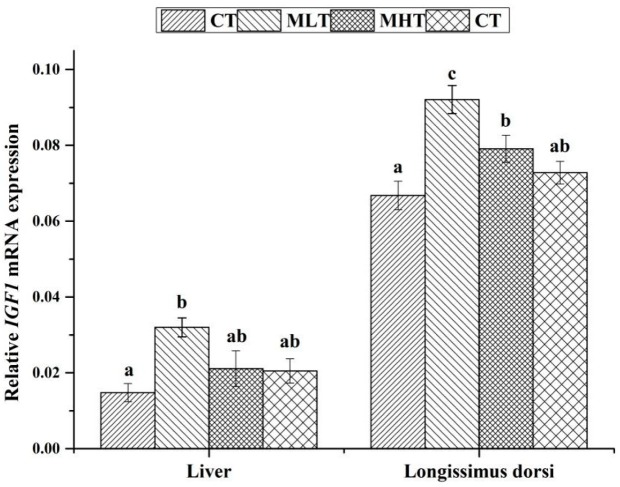
Effects of MLPs on *IGF1* mRNA expression in liver and muscle longissimus in weaning pigs. Vertical bars represent standard errors, *N* = 5. Values are expressed in means. Mean values with different letters (^a, b, c^) are significantly different within a cluster of bars, not across clusters of bars (*P* < 0.05).

Up to a certain extent, the dietary supplementation of MLPs increased the expression levels of *IGF1* mRNA in liver and muscle tissues. The results obtained after the expression analysis are shown in Figure 2. In contrast to the CT group, the expression levels of *IGF1* mRNA were higher in liver and muscle longissimus in the animals belonging to the MLT and MHT groups. The difference in the levels of *IGF1* mRNA expression was found significant (*P* < 0.05) in both liver and muscle longissimus between the MLT and CT groups. While the expression level of *IGF1* mRNA in muscle longissimus from the MLT group was significantly higher than that of the AT group (*P* < 0.05), the level of *IGF1* mRNA expression in muscle longissimus from the MHT group was substantially pronounced (*P* < 0.05) compared with the CT group. The difference in *IGF1* mRNA abundance in liver and muscle longissimus between the MHT and AT groups was insignificant (*P* > 0.05).

## 4. Discussion

After weaning, piglets lose the supply of maternal antibodies, and the development of their immune system remains incomplete. Thus, weanling pigs are particularly vulnerable to the invasion of exogenous pathogenic bacteria and viruses, leading to diarrhea, growth retardation, and even death [23]. The growth of animals is subjected to the combined effects of a progression of anabolism and catabolism. This includes the body’s gluconeogenesis, accumulation and consumption of amino acids and fatty acids, protein biosynthesis, and the regulatory effects of the immune system [24]. Animals’ immune systems contain peripheral immune organs (lymph nodes, spleen), as well as the central immune organs (bone marrow, thymus), where the immune cells mature and proliferate [17]. The status of immune organs determines the level of the body’s immunity. Our results revealed that MLP-supplemented diet improved the ADG and F/G in weanling pigs. It was also found that the dietary supplementation of MLPs (MLT or MHT) could significantly increase the immune organ weights of weanling pigs. The findings were comparable with the results of Li [25] and Hou et al. [12], which showed that MLPs improved the immune organ weight in mice. These data demonstrate that dietary supplementation of MLPs might enhance the digestion and absorption of nutrients in weanling pigs, enhance their immunity, improve the animals’ disease resistance, and reduce the inhibitory effect on metabolic processes [12,25], thereby elevating the growth performance of weanling pigs. Our results opened new avenues for improving the health of weanling pigs and reducing diet consumption.

Immunoglobulins are a class of globulins with antibody activity or antibody-like chemical structures. These are universally present in the blood, tissue fluid, and exocrine fluid. Among them, IgG, IgA, and IgM exist in the serum of all mammals, forming important indicators that reflect the body’s humoral immunity [26]. IL-l*β* is an acute inflammatory cytokine synthesized and secreted mainly by mononuclear macrophages. It is widely involved in the process and regulation of infection, stress, hypoxia, and reoxygenation. IL-2 is a peptide immune regulator secreted by activated T lymphocytes [27]. It not only promotes T-cell proliferation and participates in T-cell apoptosis, but also exerts an immunosuppressive effect [17,28]. IL-6 is mainly produced by lymphocytes and monocytes that exhibit a wide range of biological and anti-inflammatory effects. It also plays key roles in immune regulation, stress response, hematopoietic stem cell differentiation, and defense mechanisms [17]. IFN-*γ* induced in response to infection displays numerous biological functions in different cells, and is thus involved in the initiation and regulation of the immune response [29]. In this study, the elevated serum levels of IgG and IgM in the MLT group indicated increased antibody formation of B-lymphocytes. The findings indicated that MLP-induced increase in serum antibodies and cytokines in weanling pigs might be achieved by improving the performance of immune organs. We also observed that the effect of high-dose MLPs (MHT group) was not as effective as that of low-dose MLPs (MLT group). Though the explicit mechanisms were unclear in this study, elevated serum levels of IL-1*β*, IL-2, IL-6, IL-8, and IFN-*γ* in weanling pigs of the MLT-, MHT-, and AT-fed groups revealed an improvement in cellular/humoral immunity in these pigs.

Lymphocyte transformation rate is an imperative indicator to evaluate the cellular immune function [30]. Lymphocyte proliferation is a normal protective physiological response of the body’s immune system after stimulation by foreign antigens, which is of great significance for the body’s combating against exogenous microbial infection [31]. Our results showed that MLPs could promote the proliferation and transformation of lymphocytes. The transformation rate of lymphocytes from weanling pigs treated with 0.6 g/kg MLPs (i.e., the MLP group) was markedly higher than in the CT and AT groups (*P* < 0.05).

Nitric oxide is a signal molecule with diverse biological activities. It is synthesized by intracellular NO synthase (NOS), including inducible NOS (iNOS) and constitutive NOC (cNOS), through oxidation and deamination of arginine. The biological activity of NO is wide-ranging and plays an important role in immune regulation [19]. It is one of the effector molecules of macrophages killing foreign pathogens in vivo; therefore, it can enhance the body’s non-specific immune function and mediate macrophage phagocytosis. In addition, NO also affects the proliferative response of T lymphocytes. The present study observed that dietary enrichment of MLPs significantly enhanced the NO production from lymphocytes of weanling pigs (*P* < 0.05). Similarly to the results of lymphocyte proliferation, the maximum effect was observed with the animals treated with low-dose MLPs (0.6 g/kg). For example, the MLT group showed superior results over the MHT group, which was in consonance with the findings of Badovinac et al. [32], who showed that the effect of NO on lymphocyte proliferation was affected by its concentration, i.e., low concentrations of NO-stimulated lymphocyte proliferation, whereas high concentrations inhibited the proliferation.

*IGF1* is a major regulatory factor of animal growth, development, and metabolism. It also mediates the growth-promoting activity of growth hormone (GH) [33]. Though it is expressed ubiquitously in various tissues and organs in animals, the liver is the main place of its expression and synthesis [34]. The expression of the hepatic *IGF1* gene is under the regulation of GH, nutrient status, etc. [33]. In extrahepatic tissues, *IGF1* shows its effects on animal growth via autocrine and paracrine mechanisms. Many reports confirmed that the abundance of *IGF1* favorably coincided with the production performance, illustrating the significance of studies on this gene in livestock production. Our results revealed that dietary supplementation of MLPs increased the expression levels of *IGF1* mRNA in liver and muscle longissimus of weanling pigs to a certain extent in comparison with the CT group. It is generally believed that *IGF1* mRNA presents mainly in the liver [34]. However, under certain stress and dietary nutrient conditions, extrahepatic tissues may also show relatively high expression of *IGF1* mRNA [35]. We also observed that muscle longissimus expressed an elevated level of *IGF1* mRNA compared with the liver, though the potential regulatory mechanism was still uncertain. In any case, the addition of MLPs increased the expression level of *IGF1* mRNA in both liver and muscle longissimus of weanling pigs. An increase in *IGF1* mRNA expression induces lymphocyte selection and differentiation as well as the production of immunoglobulins [36], hence indirectly improving the animals’ immune function, growth performance, and resistance to diseases. This was consistent with the results of growth performance in this study, suggesting that MLPs have the function of promoting growth performance, immunity, and disease resistance in weanling pigs.

## 5. Conclusions

This report evaluated the effects of MLPs on immune functions—such as serum immunoglobulins and cytokines, lymphocyte proliferation and nitric oxide production, *IGF1* mRNA expression, etc.—in weanling pigs. The results demonstrated that the dietary supplementation of MLPs in weanling pigs elevated the levels of serum cytokines IL-1, IL-2, IL-6, IL-8, and IFN-*γ* and increased lymphocyte transformation rate and nitric oxide production. In addition, *IGF1* mRNA expression was enhanced in liver and muscle tissues. In conclusion, dietary supplementation with MLPs could improve animals’ metabolisms and the immune parameters of weanling pigs.

## Figures and Tables

**Table 1:** Chemical composition and ingredients of the basal diet.

Ingredients	Content	Nutrition Composition	Content
Maize [%]	60.50	Metabolizable energy [MJ/kg]	14.20
Fish Meal [%]	3.50	Crude protein [%]	18.75
Soybean Meal [%]	26.00	Total Ca [%]	0.91
Soybean Oil [%]	2.00	Total P [%]	0.61
Whey Powder [%]	5.00	Digestible Lys [%]	1.21
Calcium Carbonate	0.55		
Di-Calcium Phosphate [%]	1.00		
L-Lys·HCl, 78% [%]	0.15		
Salt [%]	0.30		
Mineral Premix [%]	1.00		

**Table 2:** Effects of dietary mulberry leaf polysaccharides (MLPs) on growth performances and immune organ indices of weanling pigs.

Parameters	*Treatment*			
	CT	MLT	MHT	AT
Growth Performance				
Initial Body Weight, g	9.45 ± 0.15	8.92 ± 0.26	9.26 ± 0.20	8.98 ± 0.16
Final Body Weight, g	17.8 ± 0.26	17.62 ± 0.35	17.77 ± 0.23	17.22 ± 0.14
ADG, g	441.0 ± 5.67 ^a^	462.81 ± 5.40 ^b^	447.8 ± 1.89 ^ab^	433.68 ± 1.90 ^a^
ADFI, g	737.59 ± 7.65	729.4 ± 10.54	736.47 ± 8.96	712.94 ± 2.70
F/G, g/g	1.670 ± 0.004 ^b^	1.576 ± 0.012 ^a^	1.640 ± 0.011 ^ab^	1.640 ± 0.012 ^ab^
Immune organ Indexes				
Thymus Index	2.59 ± 0.15 ^a^	2.94 ± 0.04 ^ab^	3.15 ± 0.15 ^b^	2.61 ± 0.08 ^a^
Spleen Index	1.69 ± 0.04 ^a^	1.90 ± 0.07 ^b^	1.90 ± 0.06 ^b^	1.87 ± 0.09 ^b^

*Treatments*: Control treatment (CT), basal diet (BD); MLP low-dose treatment (MLT), 0.6 g/kg MLPs + BD; MLP high-dose treatment (MHT), 1.2 g/kg MLPs + BD; antibiotic treatment (AT), 0.15 g/kg aureomycin + BD. Values are expressed as means ± SEM, *N* = 5; ^a, b^ Means with different superscripts differ significantly (*P* < 0.05).

**Table 3:** Effects of MLPs on the concentrations of immunoglobins and cytokines in weaning pigs’ serum.

Parameters	*Treatment*			
	CT	MLT	MHT	AT
IgM [mg/mL]	0.64 ± 00.03 ^ab^	0.68 ± 00.06 ^ab^	0.55 ± 00.01 ^a^	0.66 ± 00.07 ^ab^
IgG [mg/mL]	3.97 ± 00.06 ^a^	4.98 ± 00.27 ^c^	3.70 ± 00.20 ^ab^	4.05 ± 00.22 ^abc^
IL-1*β* [pg/mL]	156.36 ± 7.47 ^a^	210.39 ± 07.76 ^b^	164.84 ± 00.67 ^a^	161.59 ± 3.36 ^a^
IL-2 [pg/mL]	352.51 ± 4.52 ^a^	450.41 ± 09.16 ^c^	415.39 ± 08.62 ^bc^	361.13 ± 5.98 ^a^
IL-6 [pg/mL]	53.80 ± 1.76 ^a^	62.02 ± 04.19 ^ab^	70.68 ± 01.56 ^bc^	57.05 ± 01.48 ^a^
IL-8 [pg/mL]	222.18 ± 9.46 ^a^	271.07 ± 10.15 ^b^	231.14 ± 11.22 ^a^	233.61 ± 13.60 ^a^
IFN-*γ* [pg/mL]	44.87 ± 0.94 ^a^	50.42 ± 01.98 ^bc^	52.73 ± 01.87 ^c^	49.71 ± 01.13 ^bc^

*Treatments*: CT, basal diet; MLT, basal diet + 0.6 g/kg MLP; MHT, basal diet + 1.2 g/kg MLP; AT, basal diet + 0.15 g/kg Aureomycin. IgM, immunoglobin M; IgG, immunoglobin G; IL-1*β*, interleukin-1*β*; IL-2, interleukin-2; IL-6, interleukin-6; IL-8, interleukin-8; IFN-*γ*, interferon-*γ*. Values are expressed as means ± SEM, *N* = 5; ^a, b, c^ Means with different superscripts differ significantly (*P* < 0.05).
